# Prevalence of Anemia and Associated Risk Factors among Pregnant Women in Semarang, Indonesia, during COVID-19 Pandemic

**DOI:** 10.4314/ejhs.v33i3.8

**Published:** 2023-05

**Authors:** Ani Margawati, Ahmad Syauqy, Aras Utami, Dea Amarilisa Adespin

**Affiliations:** 1 Department of Nutrition Science, Faculty of Medicine, Diponegoro University; 2 Department of Public Health and Prevention, Faculty of Medicine, Diponegoro University

**Keywords:** anemia, pregnant women, Indonesia, COVID-19

## Abstract

**Background:**

The coronavirus disease-2019 (COVID-19) pandemic has caused several changes that affect overall health, including the prevalence of anemia in pregnant women. Several risk factors, including iron deficiency during pregnancy, diabetes, maternal smoking, preterm birth, low birth weight, and multiple pregnancies, can influence poor iron intake in infants. This study aims to analyze the prevalence and factors associated with anemia in pregnant women during the COVID-19 pandemic.

**Methods:**

A cross-sectional study was conducted on 238 pregnant women from two districts in Semarang, Indonesia. The study population was selected using a cluster sampling technique. Trained enumerators collected data through interviews using the Semi-Quantitative Food Frequency Questionnaire (SQ-FFQ) to estimate participants' food intake and anthropometric measurements. Additionally, hemoglobin levels were measured by trained nurses during antenatal care (ANC) visits. Univariate and multivariate analyses were performed using logistic regression to estimate the factors associated with anemia in pregnant women during the COVID-19 pandemic.

**Results:**

Among all participants, 14.3% (n=34) were anemic, with 32.3% and 67.6% having moderate and mild anemia, respectively. Moreover, study variables such as less compliance with ANC (antenatal care) guidelines (p = 0.020), excessive phosphorus intake (p = 0.039), inadequate zinc intake (p = 0.003), and inadequate calcium intake (p = 0.043) were associated with anemia among pregnant women.

**Conclusion:**

Anemia among pregnant women in Semarang, Indonesia, is a mild public health problem. Less compliance with ANC guidelines, excessive phosphorus intake, and inadequate zinc intake are significantly associated with anemia among pregnant women during the COVID-19 pandemic.

## Introduction

Anemia during pregnancy is a prevalent issue worldwide, affecting both developed and developing countries. This condition has serious health consequences and is linked with increased morbidity, mortality, poor birth outcomes, and impaired child development (1,2). Adverse effects of anemia during pregnancy include pre-eclampsia, premature rupture of membranes, low birth weight, preterm delivery, and maternal and fetal mortality (3,4). In a study conducted by Chaparro, it was estimated that 32.9% of the global population was anemic. Furthermore, the World Health Organization (WHO) reported that 29% of women of childbearing age and 38% of pregnant women aged 15-29 years suffered from anemia worldwide in 2011 (5). In Indonesia, according to the 2018 Basic Health Research (*Riskesdas*) data, the prevalence of anemia among pregnant women was 48.9%.

Iron deficiency is a frequently encountered complication during pregnancy, impacting approximately 22% of women in the second and third trimesters. Iron is critical in the development of organ systems, particularly the brain. Inadequate iron consumption in infants is attributable to various risk factors, such as iron insufficiency during pregnancy, maternal diabetes, smoking, preterm birth, low birth weight, and multiple pregnancies. Furthermore, the health status, nutrient intake, stress levels, and mental state of the mother during pregnancy are key factors that can influence the health and well-being of the infant (4).

The issue of mitigating the elevated prevalence of anemia among pregnant women in developing nations, including Indonesia, remains a pressing priority. The World Health Organization (WHO) has set an objective of reducing the prevalence of anemia among women of reproductive age by 50% by 2025. As a result, the Indonesian government has launched multiple initiatives to combat anemia, including the distribution of blood-boosting supplement tablets (6). Nonetheless, the ongoing COVID-19 pandemic has induced significant social transformations that have affected overall health status, including anemia, and the feasibility of implementing health programs sustainably (7,8).

The Indonesian government has enforced social restriction regulations to combat the spread of COVID-19. However, the execution of such policies has negatively impacted individuals due to income losses, including job loss, unemployment, or layoffs, which further impact the household economy. This pandemic has increased the risk of elevated anemia rates among pregnant women, particularly due to the irregular consumption of blood-boosting tablets and weakened economic conditions that could cause maternal nutritional intake to decrease. Pregnant women from low-income families are particularly vulnerable to decreased access to healthy food, heightened food insecurity, long-term uncertainty in securing employment, and reduced physical activity (9). Additionally, a prior study reported a decrease in the distribution of blood-boosting supplement tablets to reduce anemia between February and April 2020 (10).

Several developing countries in Asia have experienced an increase in the incidence of anemia because of disruptions in food supply systems and economic activities during the COVID-19 pandemic (11). These increases highlight the urgent need for local surveys to assess the prevalence of anemia among pregnant women and identify risk factors to evaluate the implementation of anemia prevention and control programs during the COVID-19 pandemic. The aim of this study is to assess the prevalence of anemia and identify the factors associated with anemia among pregnant women during the COVID-19 pandemic. The target population of this study includes pregnant women who have made antenatal care (ANC) visits during the COVID-19 pandemic, as access to health facilities, including access to adequate nutrition, has been limited.

## Methods

This study utilized an observational, cross-sectional design to examine the prevalence of anemia and associated factors among pregnant women residing in two districts of Semarang, Central Java, Indonesia. Data was collected at public health centers during antenatal care visits using systematic sampling. Two districts, Mijen and Srondol, in Semarang City were selected to represent rural-urban areas and city centers in Semarang.

Pregnant women who completed ANC visits at these health centers were systematically selected to participate in the study. Interviews were conducted after ANC visits with pregnant women who were residents of the sub-district area since the beginning of 2020 and provided written informed consent from August to September 2020. The minimum sample size of 216 pregnant women was determined using the Lemeshow formula (1997), and 238 pregnant women were ultimately included in the study, all of whom completed the measurements. The inclusion criteria included being a resident of the sub-district area since the beginning of 2020 and willingness to participate in the study by providing written informed consent. Pregnant women with pre-existing diseases at the time of data collection were excluded from the study.

The study preparation was initiated by visiting the *Puskesmas* (community health center) and meeting with the head of the targeted *Puskesmas* to obtain approval for participation in the study. Data collection was conducted through face-to-face interviews by 13 trained enumerators, who performed anthropometric measurements cautiously to minimize bias. Body weight was measured using a digital scale, while height was measured using a stadiometer. Trained nurses performed laboratory examinations, specifically hemoglobin tests.

Each participant was interviewed using a structured questionnaire to fulfill the objectives of the study. The questionnaire comprised of four sections. The first section aimed to investigate socio-demographic factors. Based on age, the study participants were categorized into three groups: pregnancy at a young age (<20 years), safe gestational age (20 — 35 years), and older age (>35 years) (12). Total incomes were categorized as low (below the minimum wage of the city) and sufficient (equal to or above the minimum wage of the city) (13). Study participants with elementary or junior high school education were classified as having low education, while participants with senior high school education or higher were classified as having moderate education (14).

The second section of the questionnaire was focused on obstetric status, in which study participants were categorized as compliant with ANC visits if they had attended at least one visit in the first trimester, one in the second trimester, and two in the third trimester (15). Additionally, gestational ages were categorized into first and second trimesters (≤28 weeks) and third trimester (>29 weeks) (16).

The third section of the questionnaire aimed to assess the medical status of the participants. Nutritional status was measured using the Mid-Upper Arm Circumference (MUAC) band. MUAC values <23.5 cm was categorized as malnutrition, while MUAC values ≥23.5 cm was categorized as a normal nutritional status (17).

In the final section of the questionnaire, information on nutritional factors was obtained. Nutritional knowledge was evaluated using a questionnaire consisting of 10 questions that were tested for validity and reliability (18,19). Participants who scored above 60 were considered to have good knowledge (20). Additionally, participants who scored above 80% were considered to have good nutritional knowledge. Food intake adequacy was assessed using the Semi-Quantitative Food Frequency Questionnaire (SQ-FFQ) and then processed using Nutri Survey. Food intake was categorized as inadequate (<90%), adequate (90 — 119%), or excessive (≥120%) (21).

Hemoglobin levels of pregnant women were measured using the Hemo Cue method. Blood samples were collected using a pipette and microcuvette, and the hemoglobin levels were measured using the Hemo Cue device. The hemoglobin levels were classified as low if they were less than 11 g/dL, and normal if they were 11 g/dL or higher (22). The severity of anemia was categorized as mild (10 — 10.9 g/dL), moderate (7 — 9.9 g/dL), and severe (less than 7 g/dL) (23).

All statistical analyses were conducted using SPSS 24 (IBM Corp., Armonk, NY, USA). Categorical variables were presented as frequencies (percentages) for all participants, including anemic and non-anemic individuals. Univariate and multivariate analyses were performed using logistic regression to estimate factors associated with anemia in pregnant women during the COVID-19 pandemic.

Pregnant women with confirmed anemia status based on hemoglobin levels were tested against predictor variables suspected to be associated with anemia.

These predictor variables were categorized into four domains: socio-demographic, obstetric status, medical status, and nutritional factors. Four multivariate-adjusted logistic regression models were used to identify independent predictor variables associated with anemia in pregnant women for each domain. An overall model that combined the four models was also employed. Variables from the final model were determined using a stepwise backward removal method, removing variables with p-values above 0.25 until an adequate model was reached. The odds ratio (OR) and 95% confidence interval (CI) were calculated for the predictor variables in the analysis. All statistical tests were two-sided, and p-values ≤ 0.05 were considered statistically significant.

This study adhered to the principles of the Helsinki Declaration (1964) for research involving human subjects. The study protocol received approval from the Ethics Committee of the Medical Faculty at Sultan Agung University, Semarang, Indonesia, under the approval number 308/IX/2020/*Komisi Bioetik*. All study participants provided written informed consent before participating in the study.

## Results

Based on the study results presented, it appears that pregnant women who had low compliance with ANC visits had a significantly higher risk of anemia during pregnancy (OR3 = 4.224, 95% CI: 1.294-13.794, p = 0.017) compared to those who had adequate compliance with ANC visits. In addition, pregnant women with inadequate zinc intake were also found to have a significantly higher risk of anemia during pregnancy (OR3 = 3.855, 95% CI: 1.301-11.427, p = 0.015) compared to those with adequate zinc intake.

It is important to note that while the overall nutrition knowledge of the pregnant women in this study was good, there were still variances in the adequacy of macronutrient and micronutrient intake from food. The fact that the majority of pregnant women had inadequate intake of energy, protein, fat, calcium, iron, and zinc is concerning as these are essential nutrients for maternal and fetal health. On the other hand, the excessive intake of fat, phosphorus, and manganese can also have negative health implications. It is crucial for pregnant women to receive proper nutrition education and guidance to ensure adequate nutrient intake and avoid excesses.

**Figure 1:**
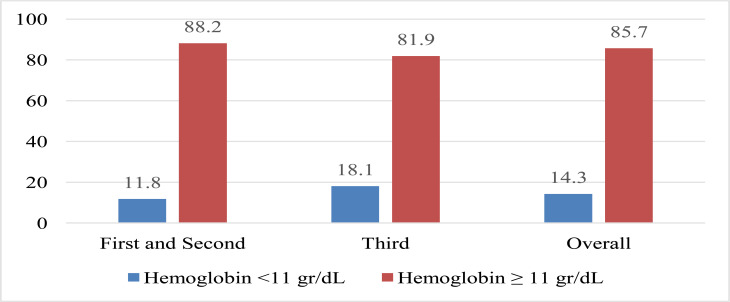
Prevalence of anemia among pregnant women according to the pregnancy trimester.

This study revealed a prevalence of 14.3% for anemia among pregnant women, as illustrated in Figure 1. Of the anemic participants, 11 (32.3%) had moderate anemia, while 23 (67.6%) had mild anemia. In terms of trimesters, the prevalence of anemia was 1.8% for the first and second trimesters and 18.1% for the third trimester.

Table 2 presents the results of the multivariate regression analyses to identify factors associated with anemia in pregnant women. We developed four separate models (model 1 for socio-demographic factors, model 2 for obstetric status, model 3 for medical status, and model 4 for nutritional factors) and an overall model (model 5) adjusting for all variables.

**Table 2:** Models of multivariate logistic analysis predicting associations between anemia and covariates in socio-demographic, obstetric, medical, and nutritional aspects among pregnant women (n=238)

Variable	aOR3)	95% CI4) for OR		*p-value*
			
		Lower	Upper	
**Model 1: Sociodemographic**				
Total income				
Sufficient (≥ minimum wage of the city)	Reference			
Low (< minimum wage of the city)	0.805	0.372	1.744	0.583
**Model 2: Obstetric status**				
ANC1) compliance				
Quite comply	Reference			
Less comply	3.994	1.212	13.158	0.023*
Gestational age				
1^st^ and 2^nd^ trimester (≤ 28 weeks)	Reference			
3^rd^ trimester (>29 weeks)	1.565	0.746	3.282	0.236
**Model 4: Nutrition factors**				
Vitamin C intake				
Excessive (≥120%).	2.054	0.373	11.328	0.409
Adequate (90 – 119%)	Reference			
Inadequate (<90 %)	3.861	0.613	24.319	0.150
Calcium intake				
Excessive (≥120%).	0.686	0.105	4.476	0.693
Adequate (90 – 119%)	Reference			
Inadequate (<90 %)	0.352	0.112	1.105	0.074
Phosphorus intake				
Excessive (≥120%).	9.135	1.123	74.339	0.039*
Adequate (90 – 119%)	Reference			
Inadequate (<90 %)	1.405	0.064	30.748	0.829
Zinc intake				
Excessive (≥120%).	1.630	0.152	17.435	0.686
Adequate (90 – 119%)	Reference			
Inadequate (<90 %)	5.924	1.850	18.968	0.003*
Manganese intake				
Excessive (≥120%).	0.941	0.098	8.998	0.958
Adequate (90 – 119%)	Reference			
Inadequate (<90 %)	10.107	0.487	209.693	0.135
**Model 5: Overall model**				
ANC1) compliance				
Quite comply	Reference			
Less comply	4.991	1.284	19.405	0.020*
MUAC2)				
Normal (≥ 23.5 cm)	Reference			
Malnutrition (< 23.5 cm)	0.370	0.101	1.358	0.134
Phosphorus intake				
Excessive (≥120%).	7.170	0.916	56.135	0.061
Adequate (90 – 119%)	Reference			
Inadequate (<90 %)	2.174	0.120	39.401	0.599
Zinc intake				
Excessive (≥120%).	0.921	0.078	10.917	0.948
Adequate (90 – 119%)	Reference			
Inadequate (<90 %)	5.430	1.671	17.647	0.005*

1) Antenatal Care

2) Mid-Upper Arm Circumference

3) adjusted Odds Ratio

4) Confidence Interval

* Data with p-value < 0.05 indicate statistically significant

In model 2, low compliance with ANC (adjusted odds ratio [aOR] = 3.994, 95% CI: 1.212-13.158, p = 0.023) independently predicted anemia among pregnant women. In model 4, excessive intake of phosphorus (aOR = 9.135, 95% CI: 1.123-74.339, p = 0.039) and inadequate intake of zinc (aOR = 5.924, 95% CI: 1.850-18.968, p = 0.003) were found to be independent predictors of anemia among pregnant women. Model 5 showed that less compliance with ANC (aOR = 4.991, 95% CI: 1.284-19.405, p = 0.020) and inadequate intake of zinc (aOR = 5.430, 95% CI: 1.671-17.647, p = 0.005) were significantly positively associated with anemia among pregnant women. On the other hand, inadequate calcium intake was found to be a significant protective factor for anemia among pregnant women (aOR = 0.298, 95% CI: 0.092-0.962, p = 0.043).

## Discussion

This study found a prevalence of 14.3% for anemia among pregnant women, indicating a mild public health problem. This prevalence is slightly lower than a previous study conducted in Semarang, Indonesia, which reported 15.82% anemia prevalence among 25,329 pregnant women. However, the prevalence in this study is much lower than the overall anemia prevalence in Indonesia, which is 48.9%. Anemia is considered a serious public health problem when it affects 40% or more of the population. Comparing the prevalence of anemia during the COVID-19 pandemic in other regions of Indonesia, the estimated prevalence in Semarang was higher than in Deli Serdang (2%), but much lower than in Samarinda (37.4%) and slightly lower than in Yogyakarta (15.8%) and Jepara (17.1%).

The results of this study indicate that pregnant women who were less compliant with ANC visits were more likely to have anemia. ANC is a crucial component of prenatal care, providing pregnant women with a range of health promotion and preventative services. The World Health Organization recommends a minimum of four ANC visits, ideally scheduled at 16, 24-28, 32, and 36 weeks of pregnancy, and emphasizes nutrition counseling as a critical component of ANC. Studies conducted in several developing countries have demonstrated that women who receive ANC services exhibit better knowledge, attitudes, and prenatal practices compared to those who do not. Nutrition education and counseling are widely employed strategies to improve the nutritional status of pregnant women, which significantly impacts fetal, infant, and maternal health outcomes.

The findings of this study were consistent with a previous study conducted in Pekanbaru, Indonesia (24). However, the current COVID-19 pandemic has led to pregnant women being hesitant to visit healthcare facilities due to fear of contracting the virus. This study found that 13 out of 238 pregnant women were less obedient to ANC visits. Previous meta-analyses conducted during the COVID-19 pandemic have also shown a decrease in antenatal care attendance in various countries, including Bangladesh, Nigeria, South Africa, and Ghana (25).

Compliance with ANC visits is essential for early detection of maternal pregnancy conditions at risk, including anemia, so that intervention problems can be addressed immediately. However, in the current Covid-19 pandemic situation, there are many restrictions on community services, including maternal and neonatal health services. Pregnant women are reluctant to go to healthcare facilities due to fear of infection, and there are suggestions to postpone pregnancy checks and classes. Additionally, there is a lack of preparedness for services in terms of personnel and infrastructure, including Personal Protective Equipment (26).

The Indonesian government has implemented a program to prevent anemia among pregnant women, which includes the provision of 90 iron tablets for each woman during pregnancy. However, despite these efforts, many pregnant women refuse or fail to comply with this recommendation due to various reasons, resulting in a high prevalence of anemia. A cross-sectional study has shown that adherence to the consumption of iron tablets was low, especially during the pandemic, as some pregnant women did not receive information on how to obtain iron tablets without having to attend ANC visits. Additionally, some respondents lacked understanding and acceptance of the side effects of iron tablets, and there was a lack of awareness about the importance of iron tablets and the dangers of anemia for pregnant women and infants (19).

ANC services are crucial for pregnant women. Similar findings were observed in a study conducted in India during the COVID-19 pandemic, where 47.1% of women in the study group did not receive regular iron and folic acid supplements during pregnancy, leading to anemia and related complications. Moreover, a study in Tanzania showed that pregnant women who received regular iron supplementation and visited ANC more or equal to four times had a lower prevalence of anemia than those with fewer ANC visits. In addition, another study demonstrated that the prevalence of anemia and severe anemia was higher during the pandemic than the pre-pandemic period.

Vitamins, minerals, and fatty acids, collectively known as micronutrients, play a crucial role in maintaining the health of pregnant women and their fetuses. Deficiencies or inadequacy of these nutrients can lead to growth and developmental deficiencies, cognitive and physiological problems, and immunodeficiencies. Hence, balanced nutrition is crucial during pregnancy, including the periconceptional period. This study used the Semi-Quantitative Food Frequency Questionnaire (SQ-FFQ) as an instrument, as it was not feasible to follow up with subjects for a 3 x 24-hour recall during the COVID-19 pandemic. The study found that the subjects consumed fish, which is a good source of phosphorus, calcium, zinc, and iron. People in Semarang, a coastal city, consume more fish than other animal protein sources such as meat and chicken. These minerals are present in food in various forms and combinations with macronutrients. Fish is a source of animal protein, essential macro minerals (calcium, phosphorus, magnesium, sodium, potassium, and chloride), and trace elements (cobalt, copper, iodine, iron, manganese, selenium, and zinc) (27).

Another study has highlighted the importance of animal source foods in preventing anemia. However, the consumption of plant-based foods can also play a critical role in reducing the risk of anemia. The study showed that iron deficiency can occur when people consume a diet low in animal-derived foods, especially when their diet is primarily composed of staple foods and when they have infections that result in blood loss or breakdown of red blood cells (28). Similarly, a cross-sectional study in the Kolaka district of Southeast Sulawesi found that during the Covid-19 pandemic, rising food prices and falling incomes caused pregnant women and their families to adjust their food consumption habits, which could have an adverse impact on maternal health. The study found that Indonesians tend to consume more carbohydrates, with less animal protein and vegetables. Moreover, worsening economic conditions have contributed to declining purchasing power, affecting the diets of families in Indonesia (19).

Iron deficiency is the most prevalent nutritional deficiency worldwide, affecting approximately 1.48 billion individuals (29). Women and young children, particularly in developing countries, are the most affected groups. Furthermore, anemia is the only nutrient deficiency with a significant prevalence in industrialized nations (30,31). Iron deficiency anemia (IDA) is linked to weakness, shortness of breath, and serious health risks, including abnormal mental and motor development. Early symptoms may include glossitis or dysphagia, although they are rare (32,33). Treating IDA is a significant public health objective, particularly in developing countries.

Iron deficiency may coexist with deficiencies of other trace elements such as zinc, which is more commonly found in developing countries. Zinc functions as a catalyst in iron metabolism in the activity of the alpha-aminolaevulinic acid dehydratase enzyme, which plays a role in heme synthesis (34). In addition, zinc is found in the structure of the growth factor independent 1B (Gfi-1B) zinc finger protein, which acts as a regulator in erythroid cell growth by modulating gene expression specific to the erythroid series (35,36,37).

This study found a significant association between anemia and inadequate zinc intake among pregnant women. This result is consistent with a previous study that reported a higher prevalence of low blood zinc levels in the anemia group compared to the control group. Another study in New Zealand also demonstrated that zinc was the only micronutrient significantly related to the risk of anemia (38). Zinc plays a crucial role as a regulator of erythroid cell growth by modulating the expression of specific genes. Furthermore, it acts as a catalyst for heme iron metabolism by being part of the Gfi-1B transcriptional repressor finger protein structure, which is the main regulator of erythroid cell growth. Zinc also affects hemoglobin through a zinc-dependent enzyme system that fights oxidative stress and maintains cell integrity. The role of zinc in iron metabolism highlights the link between inadequate zinc intake and the incidence of anemia (38).

Additionally, this study has demonstrated that anemia was associated with excessive phosphorus intake among the participants. This finding was consistent with previous studies, which have shown that a high level of body phosphorus is linked to mild and moderate anemia (40) Phosphorus acts as an inhibiting factor in the production of red blood cells. Hence, hyperphosphatemia is associated with inflammation and can affect normal cellular physiology, including erythropoiesis. Moreover, high levels of phosphorus can lead to vascular calcification in the renal arteries, resulting in erythropoietin deficiency and anemia (39).

This study has shown that there is an association between inadequate calcium intake and anemia. During pregnancy, calcium absorption in the body increases, and therefore, pregnant women need to maintain adequate calcium intake, which is not significantly different from the general adult population (1200 mg/day) (40). Calcium plays a crucial role in reducing adverse outcomes and the risk of hypertension during pregnancy, which is associated with maternal deaths and a considerable risk of premature birth, the leading cause of early neonatal and infant mortality. Calcium adequacy is especially vital during the third trimester to meet the needs of the rapidly mineralized fetal skeleton. Poor pre-pregnancy bone mineral density and low calcium and vitamin D intake during pregnancy can increase the risk of low bone mass and osteoporosis in the future (41). However, excessive calcium consumption may increase the risk of urinary stones and urinary tract infections and reduce the absorption of other micronutrients (40). It is important to note that calcium is known as an inhibiting factor for iron absorption. Therefore, consuming too much calcium may reduce total absorbed iron, primarily by reducing the initial absorption of heme iron (30).

This study had several limitations that need to be acknowledged. Firstly, data collection was only conducted at the community health center (*Puskesmas*) due to the COVID-19 pandemic, and home visits to the residents of pregnant mothers were unfeasible. This may have resulted in the exclusion of pregnant women who did not visit the public health center for antenatal care, which may have affected the generalizability of the findings. Secondly, the cross-sectional design of this study did not allow for the establishment of causality or the direction of the relationship between the variables. Thirdly, the hemoglobin analysis method used in this study did not employ a standard automatic analyzer, which may have affected the accuracy of the results. Lastly, there may have been other confounding variables that were not measured in this study that could have influenced the results.

To reduce bias, trained enumerators were employed for data collection to ensure that the data obtained were in accordance with the study objectives. Despite the limitations, the findings of this study may contribute to reducing anemia among pregnant women in developing countries. Future studies should consider addressing the limitations mentioned in this study and employ a more robust study design to establish the causality and generalizability of the findings.

In conclusion, these are all important recommendations based on the findings of the study. Pregnant women should be educated on the importance of adequate nutrition during pregnancy, especially with regard to zinc, calcium, and iron intake. Health providers should also emphasize the importance of ANC compliance to monitor the health of both the mother and fetus.

It is also important to note that access to nutritious foods may be a challenge for some pregnant women, particularly those in lower socioeconomic groups. Therefore, efforts to improve food security and provide nutritional support to pregnant women should also be considered. Additionally, further research on the risk factors for anemia in different settings can provide more comprehensive and deeper insights to address this issue.

## Figures and Tables

**Table 1:** Characteristics of study participants

Variable	Total 238 (100)	Anemia 34 (14.3)	OR3)	95% CI4) for OR		*p-value*

				Lower	Upper	
**Socio-demographic**						
Age						
20-35 years	36 (15.1)	4 (11.8)	Reference			
>35 years	198 (83.2)	29 (85.3)	0.728	0.240	2.214	0.576
<20 years	4 (1.7)	1 (2.9)	1.943	0.195	19.321	0.571
Total income						
Sufficient	151 (63.5)	23 (67.7)	Reference			
Low	87 (36.5)	11 (32.4)	0.805	0.372	1.744	0.583
Education						
Moderate	189 (79.4)	28 (82.4)	Reference			
Low	49 (20.6)	6 (17.6)	0.802	0.312	2.062	0.647
ANC1) compliance						
Quite comply	225 (94.5)	29 (85.3)	Reference			
Less comply	13 (5.5)	5 (14.7)	4.224	1.294	13.794	0.017*
Gestational age						
1^st^ and 2^nd^ trimester	144 (60.5)	17 (50.0)	Reference			
3^rd^ trimester	94 (39.5)	17 (50.0)	1.649	0.795	3.421	0.179
**Nutritional Factors**						
Nutritional knowledge						
Good	228 (95.8)	33 (97.1)	Reference			
Low	10 (4.2)	1 (2.9)	0.657	0.081	5.354	0.694
Calory intake						
Excessive	68 (28.6)	9 (26.5)	0.702	0.286	1.720	0.439
Adequate	84 (35.3)	15 (44.1)	Reference			
Inadequate	86 (36.1)	10 (29.4)	0.605	0.255	1.436	0.255
Protein intake						
Excessive	25 (10.5)	2 (5.9)	0.383	0.077	1.895	0.239
Adequate	54 (22.7)	10 (29.4)	Reference			
Inadequate	159 (66.8)	22 (64.7)	0.707	0.311	1.606	0.407
Fat intake						
Excessive	78 (32.8)	13 (38.2)	1.145	0.478	2.746	0.761
Adequate	74 (31.1)	11 (32.4)	Reference			
Inadequate	86 (36.1)	10 (29.4)	0.754	0.301	1.889	0.546
Vitamin C intake						
Excessive	157 (66.0)	23 (67.6)	2.146	0.476	9.680	0.321
Adequate	27 (11.3)	2 (5.9)	Reference			
Inadequate	54 (22.7)	9 (26.5)	2.500	0.501	12.486	0.264
Calcium intake						
Excessive	12 (5.0)	2 (5.9)	0.700	0.119	4.104	0.693
Adequate	27 (11.4)	6 (17.6)	Reference			
Inadequate	199 (83.6)	26 (76.5)	0.526	0.194	1.425	0.206
Phosphorus intake						
Excessive	186 (78.2)	32 (94.2)	7.065	0.933	53.309	0.058
Adequate	35 (14.7)	1 (2.9)	Reference			
Inadequate	17 (7.1)	1 (2.9)	2.125	0.125	36.182	0.602
Iron intake						
Excessive	6 (2.5)	2 (5.9)	2.500	0.370	16.888	0.347
Adequate	36 (15.1)	6 (17.6)	Reference			
Inadequate	196 (82.4)	26 (76.5)	0.765	0.290	2.015	0.587
Zinc intake						
Excessive	12 (5.1)	1 (2.9)	1.523	0.155	14.920	0.718
Adequate	71 (29.8)	4 (11.8)	Reference			
Inadequate	155 (62.1)	29 (85.3)	3.855	1.301	11.427	0.015*
Manganese intake						
Excessive	225 (94.5)	31 (91.2)	1.278	0.154	10.577	0.820
Adequate	9 (3.8)	1 (2.9)	Reference			
Inadequate	4 (1.7)	2 (5.9)	8.000	0.459	139.290	0.154

1) Antenatal Care

2) Mid-Upper Arm Circumference

3) Odds Ratio

4) Confidence Interval

* Data with p-value < 0.05 indicate statistically significant
